# A Novel Probiotic *Bacillus subtilis* Strain Confers Cytoprotection to Host Pig Intestinal Epithelial Cells during Enterotoxic *Escherichia coli* Infection

**DOI:** 10.1128/spectrum.01257-21

**Published:** 2022-06-23

**Authors:** Sudhanshu Sudan, Xiaoshu Zhan, Julang Li

**Affiliations:** a Department of Animal Biosciences, University of Guelph, Guelph, Ontario, Canada; University of Nevada—Reno

**Keywords:** antimicrobial, enterotoxic *E. coli*, extreme environment, *Bacillus subtilis*, antimicrobial agents, cytoprotection, probiotic

## Abstract

Enteric infections caused by enterotoxic Escherichia coli (ETEC) negatively impact the growth performance of piglets during weaning, resulting in significant economic losses for the producers. With the ban on antibiotic usage in livestock production, probiotics have gained a lot of attention as a potential alternative. However, strain specificity and limited knowledge on the host-specific targets limit their efficacy in preventing ETEC-related postweaning enteric infections. We recently isolated and characterized a novel probiotic Bacillus subtilis bacterium (CP9) that demonstrated antimicrobial activity. Here, we report anti-ETEC properties of CP9 and its impact on metabolic activity of swine intestinal epithelial (IPEC-J2) cells. Our results showed that pre- or coincubation with CP9 protected IPEC-J2 cells from ETEC-induced cytotoxicity. CP9 significantly attenuated ETEC-induced inflammatory response by reducing ETEC-induced nitric oxide production and relative mRNA expression of the Toll-like receptors (TLRs; TLR2, TLR4, and TLR9), proinflammatory tumor necrosis factor alpha, interleukins (ILs; IL-6 and IL-8), augmenting anti-inflammatory granulocyte-macrophage colony-stimulating factor and host defense peptide mucin 1 (MUC1) mRNA levels. We also show that CP9 significantly (*P *<* *0.05) reduced caspase-3 activity, reinstated cell proliferation and increased relative expression of tight junction genes, claudin-1, occludin, and zona occludens-1 in ETEC-infected cells. Finally, metabolomic analysis revealed that CP9 exposure induced metabolic modulation in IPEC J2 cells with the greatest impact seen in alanine, aspartate, and glutamate metabolism; pyrimidine metabolism; nicotinate and nicotinamide metabolism; glutathione metabolism; the citrate cycle (TCA cycle); and arginine and proline metabolism. Our study shows that CP9 incubation attenuated ETEC-induced cytotoxicity in IPEC-J2 cells and offers insight into potential application of this probiotic for ETEC infection control.

**IMPORTANCE** ETEC remains one of the leading causes of postweaning diarrhea and mortality in swine production. Due to the rising concerns with the antibiotic use in livestock, alternative interventions need to be developed. In this study, we analyzed the cytoprotective effect of a novel probiotic strain in combating ETEC infection in swine intestinal cells, along with assessing its mechanism of action. To our knowledge, this is also the first study to analyze the metabolic impact of a probiotic on intestinal cells. Results from this study should provide effective cues in developing a probiotic intervention for ameliorating ETEC infection and improving overall gut health in swine production.

## INTRODUCTION

Maintenance of a healthy gastrointestinal tract is very critical in animal production. Due to the underdeveloped gut at weaning, animals are very susceptible to enteric infections (1). Enterotoxic Escherichia coli (ETEC) remains one of the leading causes of postweaning diarrhea in swine production rendering significant economic losses to swine industry (2). Due to the increase in antibiotic resistance across the globe, the use of antibiotics in animal agriculture has been seriously criticized, resulting in a ban on their use in the majority of the developed world. More recently, alternatives such zinc oxide have also undergone serious scrutiny due to their negative environmental impact and influence on the development of antibiotic-resistant microorganisms, ultimately resulting in the decision to prohibit their use in Europe (3, 4). Characterization and development of novel methods of interventions are therefore needed for disease control in animal production.

Direct-fed microbes or probiotic bacteria have been studied extensively for this purpose and have gained a lot of attention due to their ability to interact with and modulate intestinal cells and the immune system (5, 6). For the use as an antibiotic alternative, whether prophylactic measures or treatment, the anti-infective properties of the probiotic bacteria are important and desirable for characterization and development. Growing evidence suggests that some probiotic strains may prevent pathogenic colonization in the intestines of the host by either competitive exclusion, direct inhibition, or both (7, 8). Competitive exclusion by probiotics can be attributed to nutrient depletion (9) or a reduction in pathogenic attachment to the intestinal walls (10). On the other hand, direct inhibition of the pathogenic bacteria has been reported via secretion of antimicrobial secondary metabolites or bactericidal toxins (11, 12). In addition, some probiotic strains can interact with the gut microenvironment and modulate physiological functions in the host. During a pathogenic infection, specific mechanisms by which probiotic strains may confer cytoprotection could be attributed to modulation of immune system via influencing inflammatory cytokines, Toll-like receptors (TLRs) (13–15) and host defense peptides (HDPs) (16), strengthening the intestinal barrier function via the modulation of tight-junction genes (17–19), reduction of stress responses such as nitric oxide (NO) (17, 20), and apoptosis (21–23). In addition, regeneration and augmentation of cell proliferation is also reported as part of their cytoprotective role in the intestines (24–26).

Even though probiotics have been researched and developed for use in animals and humans, mixed success has been obtained practically in combating enteric infections (5). This could be attributed to strain/species specificity, efficacy, and lack of understanding of their interactions with host cells (27–31). Recently, advancement in the molecular and mass spectrophotometric techniques has facilitated the assessment and characterization of diverse complexity in host-probiotic interaction (5). This is particularly important since metabolic influence of the probiotic microbes is largely driven by their evolutionary and ecological cues (32–36), which could directly impact host physiological functions. The metabolomic influence of probiotic bacteria on hosts remains an underexplored area of research, and assessing the metabolomic profiles of these interactions may therefore provide functional cues to the development of a host-tailored next generation of probiotics. We recently isolated a novel Bacillus subtilis strain (CP9) from sub-Saharan camel feces, characterized this strain in our lab (37), and showed that CP9 had excellent probiotic properties, including a broad-range antibacterial effect that was independent of toxin secretion (38). We also showed that CP9 caused metabolic dysregulation in ETEC. Here, we test the hypothesis that CP9 can confer cytoprotection to IPEC-J2 cells during an experimental ETEC infection *in vitro.* We evaluate the ability of CP9 to attenuate ETEC infection in IPEC-J2 cells by performing pre- and coincubation assays. We further characterize the mode of action of the CP9 in IPEC-J2 cells and decipher the metabolic impact that CP9 has on IPEC-J2 cells.

## RESULTS

### CP9 inhibits ETEC from infecting IPEC-J2 cells.

**FIG 1:**
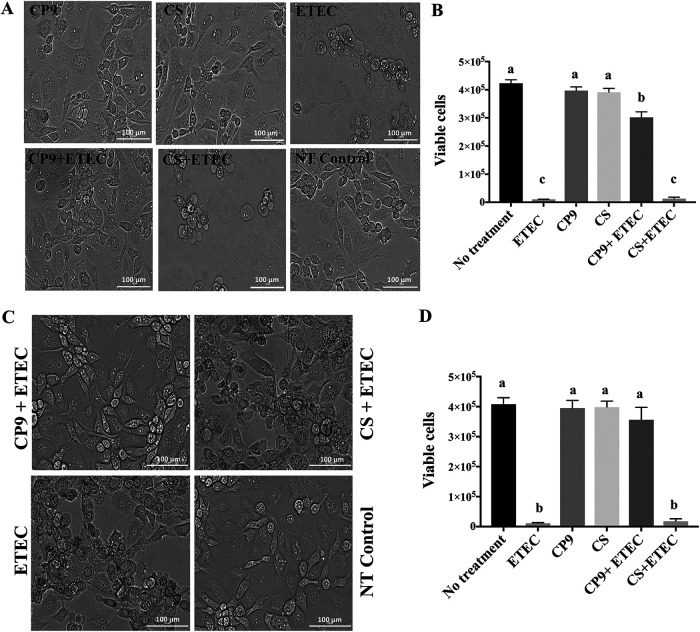
Impact of CP9 on ETEC-induced cytotoxicity in IPEC-J2 cells. (A) Change in cell morphology seen via preincubation assay. (B) Viability of IPEC-J2 cells after preincubation assay determined by trypan blue staining. (C) Change in cell morphology seen via coincubation assay. (D) Viability of IPEC-J2 cells after coincubation assay determined by trypan blue staining. The morphology of the cells was captured by using a Cytation 5 Cell Imaging Multi-Mode Reader at ×20 magnification, with a 100-μm scale in bright-field mode. The data are presented as means ± the SEM. Means marked with different letters (a, b, and c) differ significantly (*P* < 0.05).

To examine the impact of CP9 in attenuating ETEC-induced cytotoxicity in IPEC-J2 cells, we performed co- and preincubation assays. No morphological changes were observed when IPEC-J2 cells were incubated with CP9 or commercially available B. subtilis, CS. However, a clear cell damage was morphologically observed for the cells incubated with ETEC (Fig. 1A and C). Our results showed that ETEC-induced cell damage was clearly attenuated when IPEC-J2 cells were simultaneously or preincubated with CP9, but not with CS (Fig. 1A and C). Furthermore, the viability of the IPEC-J2 cells was not affected by the incubation with CP9 or CS; however, ETEC caused significant (*P *<* *0.0001) IPEC-J2 cell death after 4 h of incubation (Fig. 1B and D). Consistently, the viability of IPEC-J2 cells infected with ETEC was significantly (*P *<* *0.0001) maintained when co- or preincubated with CP9, but not with CS (Fig. 1B and D). Combined, these results suggest that CP9 protects IPEC-J2 cells from ETEC-induced toxicity and cell damage.

### CP9 reduces ETEC attachment to IPEC cells.

**FIG 2:**
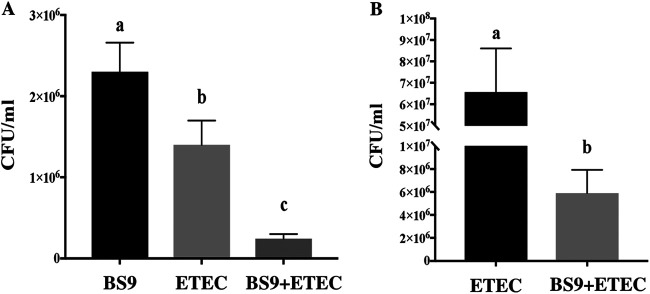
Inhibitory effect of CP9 on ETEC adhesion to IPEC-J2 cells. (A) Bacterial cell concentration determined in cell surface adhesion assay. (B) ETEC cell concentration determined in spent media. The data are presented as means ± the SEM. Means marked with different letters (a, b, and c) differ significantly (*P* < 0.05).

To investigate the inhibitory effect of CP9 on ETEC adhesion to the IPEC-J2 cells, we performed a coincubation assay, where equal numbers of CP9 and ETEC were incubated with IPEC-J2 cells for 3 h at 37°C. CP9 monoculture showed higher adhesion to the intestinal cells than ETEC monoculture, and CP9 significantly (*P *<* *0.05) reduced the ETEC adhesion to IPEC-J2 cells when incubated together with ETEC (Fig. 2A). In addition, analysis of the viable ETEC in the spent media showed significantly (*P *<* *0.05) lower ETEC counts compared to IPEC-J2 cells incubated with ETEC alone (Fig. 2B). These results suggest that CP9 may inhibit ETEC adhesion to IPEC-J2 cells by competitively acquiring the binding sites on IPEC-J2 cells and also reducing ETEC growth in a coculture.

### CP9 moderates ETEC-induced nitric oxide production in IPEC-J2 cells.

**FIG 3:**
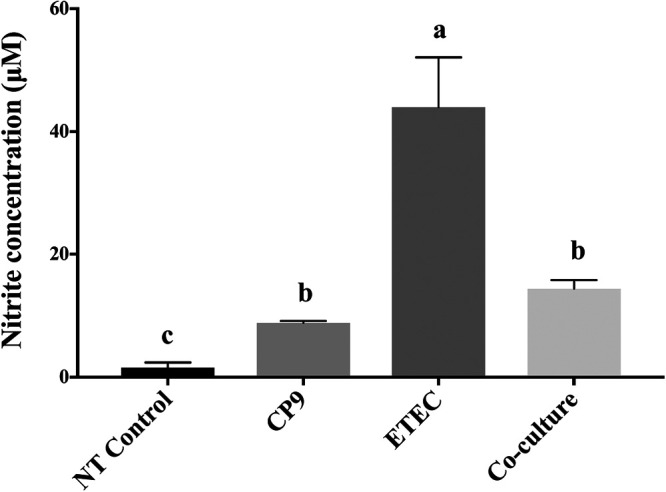
Nitric oxide production in IPEC-J2 cells. The impact of CP9 on nitric oxide production in ETEC-induced cells was assessed by calculating the nitrite concentration by using a Griess assay. The data are presented as means ± the SEM. Means marked with different letters (a, b, and c) differ significantly (*P* < 0.05).

To analyze the ability of the probiotic in modulating NO production in IPEC-J2 cells during ETEC infection, we performed a coculture assay. Our results showed that ETEC induced significantly higher (*P *<* *0.01) levels of NO production in IPEC-J2 cells compared to the no-treatment control and CP9-treated cells (Fig. 3). In addition, NO production by CP9 was significantly higher (*P *<* *0.05) than the no-treatment control cells. Interestingly, the results showed that when coincubated with ETEC, CP9 reduced the NO production in the IPEC-J2-infected cells, which was still significantly (*P *<* *0.01) higher than for the no-treatment control cells. Combined, these results suggest that CP9 may exhibit its cytoprotective role in IPEC-J2 cells by moderating the NO production in the cells.

### CP9 exhibits immunomodulatory activity in ETEC-infected IPEC-J2 cells.

**FIG 4:**
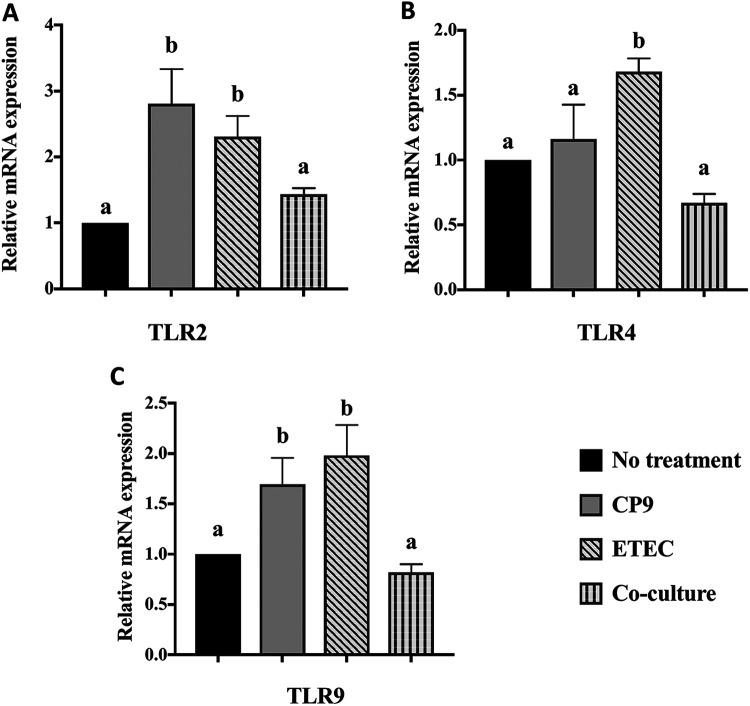
Relative gene expression of Toll-like receptors in IPEC-J2 cells. A coincubation assay was performed for 4 h with equal numbers of CP9 and ETEC in IPEC-J2 cells. The relative expression of TLR2 (A), TLR4 (B), and TLR9 (C) mRNAs was calculated by real-time PCR. The expression levels of all target genes were calculated relative to the housekeeping gene GAPDH using the 2^−ΔΔ^*^CT^* method. The values for the control cells were set to 1. The data are presented as means ± the SEM. Means marked with different letters (a, b, and c) differ significantly (*P* < 0.05).

To understand the impact of CP9 on inflammatory responses induced by ETEC-infected IPEC-J2 cells, we first analyzed the relative expression of mRNAs for selected genes encoding the TLRs (Fig. 4). Compared to no-treatment control, incubation with CP9 alone significantly (*P *<* *0.05) increased the relative expression of TLR2 and TLR9 mRNAs but not TLR4 mRNAs. As expected, the relative expression of TLR2, TLR4, and TLR9 mRNAs significantly (*P *<* *0.05) increased in the ETEC-infected cells. The relative expression of TLR2, TLR4, and TLR9 mRNAs was significantly (*P *<* *0.05) lower in cells coincubated with CP9 and ETEC. Interestingly, incubation with CP9 lowered the relative expression of the TLR2, TLR4, and TLR9 mRNAs to the negative-control levels in ETEC-infected cells, indicating a homeostatic expression of the TLRs.

**FIG 5:**
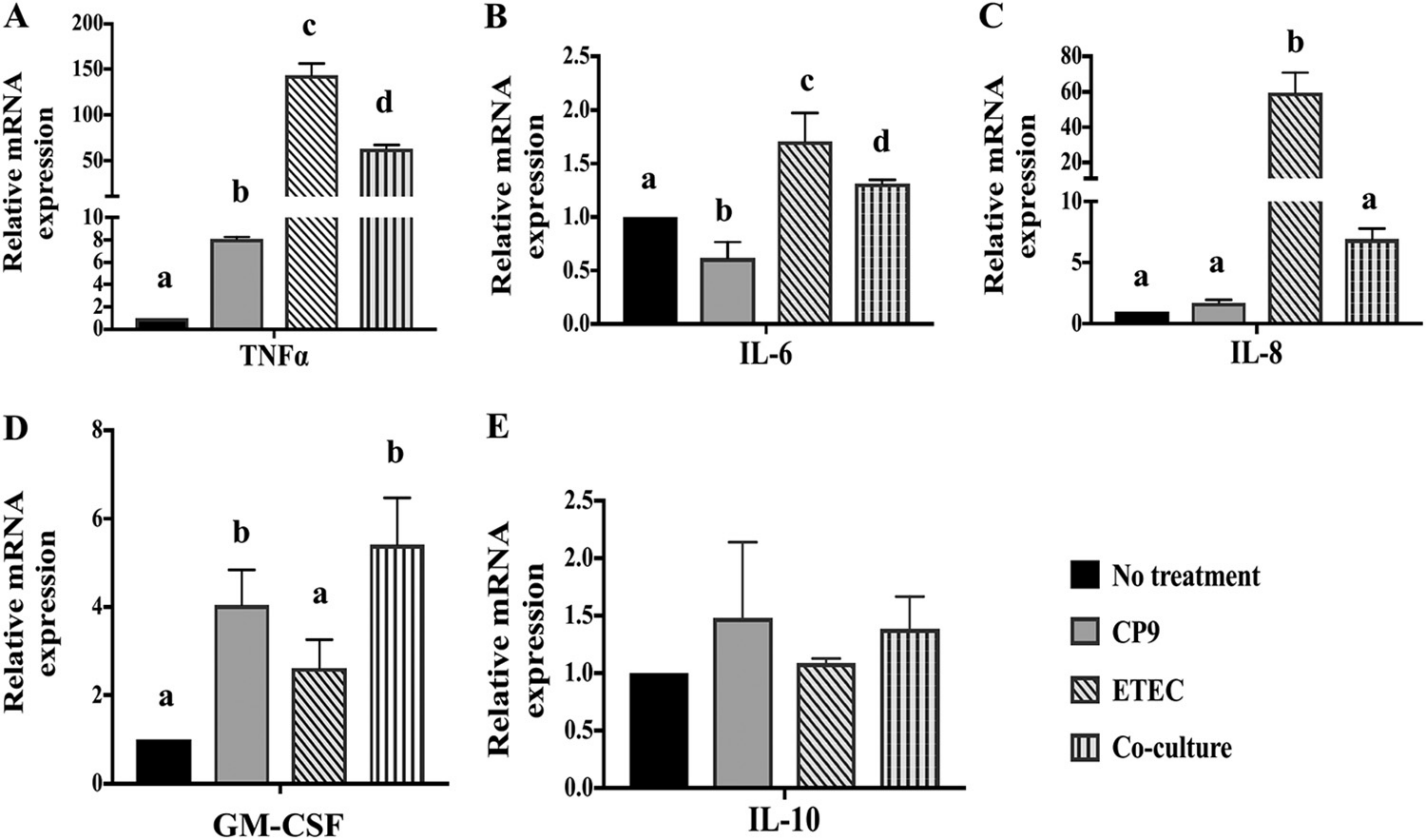
Relative gene expression of anti- and proinflammatory cytokines in IPEC-J2 cells. A coincubation assay was performed for 4 h with equal numbers of CP9 and ETEC in IPEC-J2 cells. The relative expression of TNF-α (A), IL-6 (B), IL-8 (C), GM-CSF (D), and IL-10 (E) mRNAs was calculated by real-time PCR. The expression levels of all target genes were calculated relative to the housekeeping gene GAPDH using the 2^−ΔΔ^*^CT^* method. The values for the control cells were set to 1. The data are presented as means ± the SEM. Means marked with different letters (a, b, and c) differ significantly (*P* < 0.05).

To test whether CP9 can attenuate the inflammatory response induced by ETEC in IPEC-J2 cells, we measured the relative expression of tumor necrosis factor alpha (TNF-α), interleukin-6 (IL-6), IL-8, granulocyte-macrophage colony-stimulating factor (GM-CSF), and IL-10 mRNAs (Fig. 5). Compared to no-treatment control, incubation with CP9 alone significantly (*P *<* *0.05) reduced the relative expression of IL-6 mRNA but not of TNF-α and IL-8 mRNA (Fig. 5A to C). As expected, infection with ETEC significantly (*P *<* *0.05) increased the levels of TNF-α, IL-6, and IL-8 mRNAs compared to no-treatment control and CP9 alone. Interestingly, this upsurge was significantly (*P *<* *0.05) attenuated when CP9 was coincubated with ETEC (Fig. 5A to C). Furthermore, incubation with CP9 alone significantly (*P *<* *0.05) increased the relative expression of GM-CSF mRNA (Fig. 5D); however, no significant change was observed in ETEC-infected cells. Similarly, in the presence of CP9, ETEC-infected IPEC-J2 cells showed a higher relative expression of GM-CSF mRNA. In addition, there was no significant difference observed in the relative levels of IL-10 mRNA when IPEC-J2 cells were incubated with CP9 alone, ETEC alone, or both in coculture (Fig. 5E).

**FIG 6:**
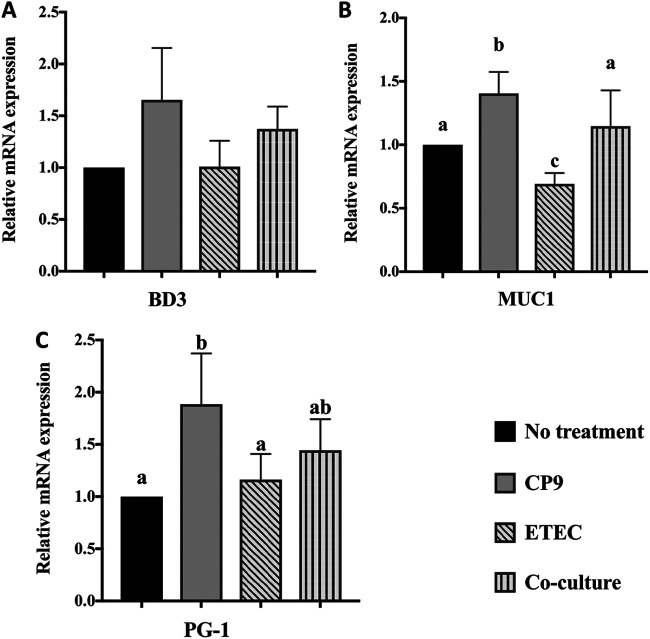
Relative gene expression of HDPs in IPEC-J2 cells. A coincubation assay was performed for 4 h with equal numbers of CP9 and ETEC in IPEC-J2 cells. The relative expression of BD3 (A), MUC1 (B), and PG-1 (C) mRNAs was calculated by real-time PCR. The expression levels of all target genes were calculated relative to the housekeeping gene GAPDH using the 2^−ΔΔ^*^CT^* method. The values for the control cells were set to 1. The data are presented as means ± the SEM. Means marked with different letters (a, b, and c) differ significantly (*P* < 0.05).

To determine whether CP9 can modulate expression of host defense peptides (HDPs), we analyzed the relative expression of BD3, MUC1, and PG-1 in the IPEC-J2 cells infected with ETEC (Fig. 6). Although an increasing trend in the relative expression of BD3 was observed in cells incubated with CP9 alone and in coculture with ETEC, the difference was not significant (Fig. 6A). Similarly, no difference in expression of BD3 was observed in the ETEC-alone cells compared to no-treatment control cells. Incubation with CP9 alone significantly (*P *<* *0.05) increased the relative expression of MUC1 mRNA (Fig. 6B). Incubation with ETEC alone significantly (*P *<* *0.05) decreased the relative expression of MUC1 mRNA, which was significantly (*P *<* *0.05) attenuated when cells were cocultured with CP9. Lastly, incubation with CP9 alone significantly (*P *<* *0.05) increased the relative expression of PG-1 mRNA (Fig. 6C); however, no significant difference was observed in PG-1 mRNA expression with other treatments. Collectively, these data suggest that CP9 has the ability to modulate the expression of immune markers by either decreasing or maintaining the homeostatic expression of inflammatory and HDP genes.

### CP9 improves the expression of intestinal barrier function genes in ETEC-infected IPEC-J2 cells.

**FIG 7:**
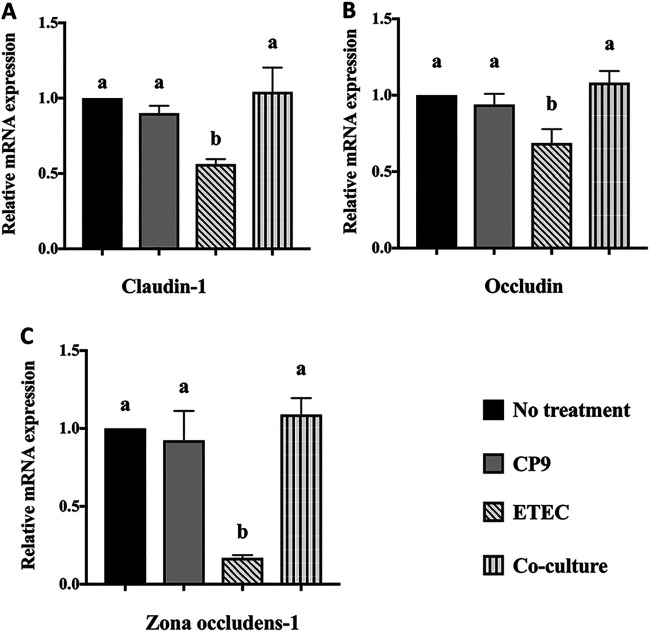
Relative gene expression of tight-junction genes in IPEC-J2 cells. A coincubation assay was performed for 4 h with equal numbers of CP9 and ETEC in IPEC-J2 cells. The relative expression of claudin-1 (A), occludin (B), and zona occludens-1 (C) mRNAs was calculated by real-time PCR. The expression levels of all target genes were calculated relative to the housekeeping gene GAPDH using the 2^−ΔΔ^*^CT^* method. The values for the control cells were set to 1. The data are presented as means ± the SEM. Means marked with different letters (a, b, and c) differ significantly (*P* < 0.05).

To test whether CP9 can attenuate the reduction in intestinal barrier function induced by ETEC in IPEC-J2 cells, we measured the relative expression of claudin-1, occludin, and tight-junction protein-1 (zona occludens-1) mRNAs (Fig. 7). Compared to control cells, incubation with CP9 alone did not demonstrate any significant change in any of the three intestinal barrier function genes tested (Fig. 7). As expected, infection with ETEC significantly (*P *<* *0.05) reduced the relative expression of claudin-1, occludin, and zona occludens-1 mRNAs (Fig. 7). Interestingly, this reduction was significantly (*P *<* *0.05) reversed in the coculture samples, where IPEC-J2 cells were coincubated with CP9 and ETEC. Combined, these results suggest ETEC reduced the mRNA expression of intestinal barrier function genes, and CP9 addition to the ETEC-infected IPEC-J2 cells may strengthen the barrier function by improving the levels of claudin-1, occludin, and zona occludens-1 mRNAs.

### CP9 reduces apoptosis in ETEC-infected IPEC J2 cells.

**FIG 8:**
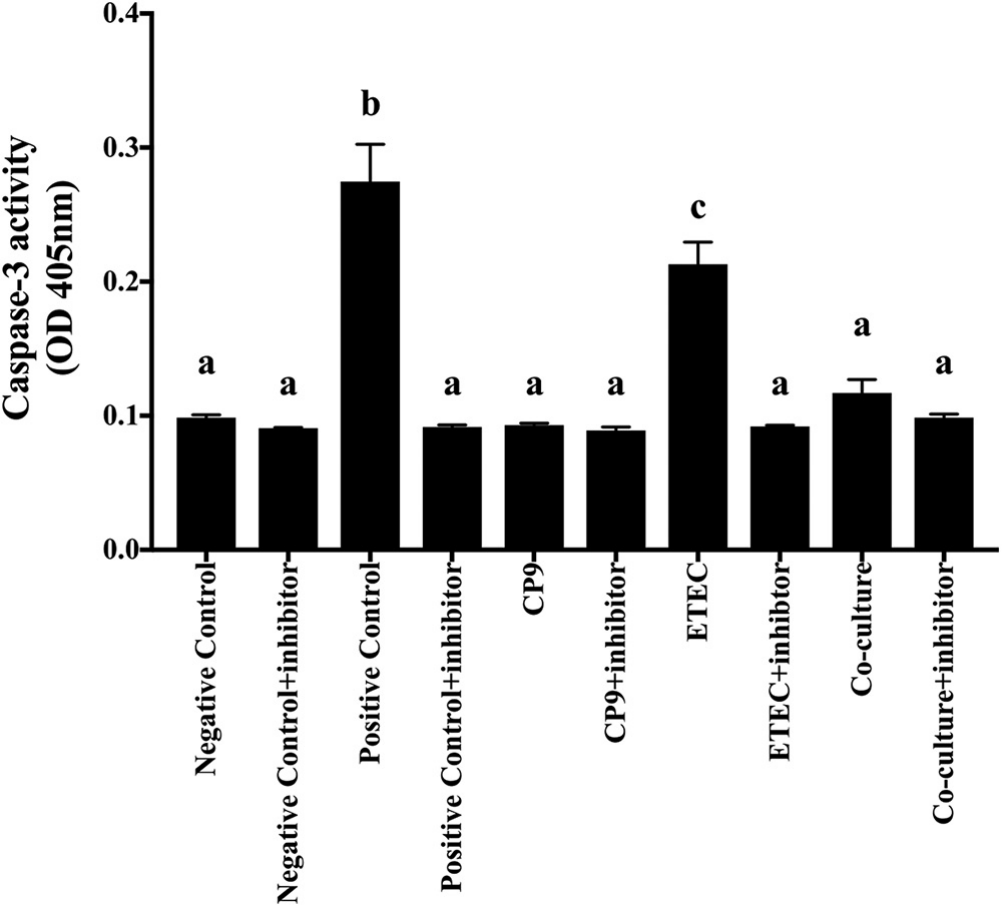
Caspase-3 activity in IPEC-J2 cells. A coincubation assay was performed for 4 h with equal numbers of CP9 and ETEC in IPEC-J2 cells. Cell lysates were collected, and the caspase-3 activity was determined by using caspase-3 colorimetric data. The data are presented as means ± the SEM. Means marked with different letters (a, b, and c) differ significantly (*P* < 0.05).

To test whether CP9 can attenuate the ETEC-induced apoptosis in IPEC-J2 cells, we measured the caspase-3 activity in IPEC-J2 cells infected with ETEC (Fig. 8). Although CP9 alone did not show any changes in caspase-3 activity compared to no-treatment control cells, the caspase-3 positive control and ETEC increased caspase-3 activity after 4 h of incubation in IPEC-J2 cells. Caspase-3 activity diminished with the addition of caspase-3 inhibitor in all treatment groups. Interestingly, caspase-3 activity was significantly (*P *<* *0.05) reduced to no-treatment control samples in the CP9- and ETEC-coincubated IPEC-J2 cells. These data suggest that CP9 may have the ability to reduce apoptosis by reducing the caspase-3 activity either directly or by reducing ETEC-induced cytotoxicity in IPEC-J2 cells.

### CP9 reinstates cellular proliferation during ETEC infection.

**FIG 9:**
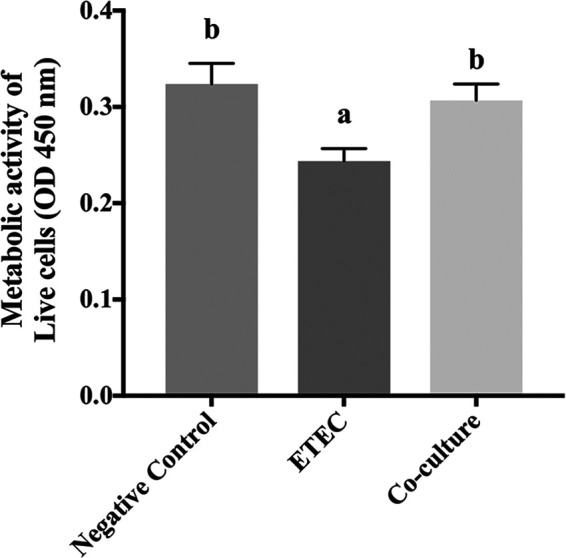
Cell proliferation analysis in IPEC-J2 cells. IPEC-J2 cells were stimulated with ETEC for 2 h and then incubated with CP9 in fresh medium for 8 h. Metabolically active cells in proliferation were counted with a CCK-8 kit by measuring the absorbance at 450 nm using a Cytation 5 Cell Imaging Multi-Mode Reader. The data are presented as means ± the SEM. Means marked with different letters (a, b, and c) differ significantly (*P* < 0.05).

Enterotoxic E. coli induces cell cycle arrest and has been shown to negatively affect intestinal cell proliferation (39–41). Since apoptosis and cell proliferation are very critical steps in epithelial cell turn over in intestinal cells (42), we next assessed whether CP9 could attenuate the impact of ETEC on IPEC-J2 cell proliferation. As expected, incubation of IPEC-J2 cells with ETEC for 2 h caused a significant decrease in IPEC-J2 cell proliferation as assessed by CCK-8 assay after 8 h (Fig. 9). The results showed that metabolic activity of cells in proliferation was significantly lower in cells incubated with ETEC than in negative-control cells. In addition, when incubated with CP9 for 8 h, ETEC-incubated IPEC-J2 cells showed a metabolic activity similar to that of negative-control cells. This suggests that CP9 was able to reinstate cellular proliferation and attenuate the negative impact of ETEC on IPEC-J2 cell proliferation.

### CP9 exposure induces metabolic modulation in IPEC J2 cells.

**FIG 10:**
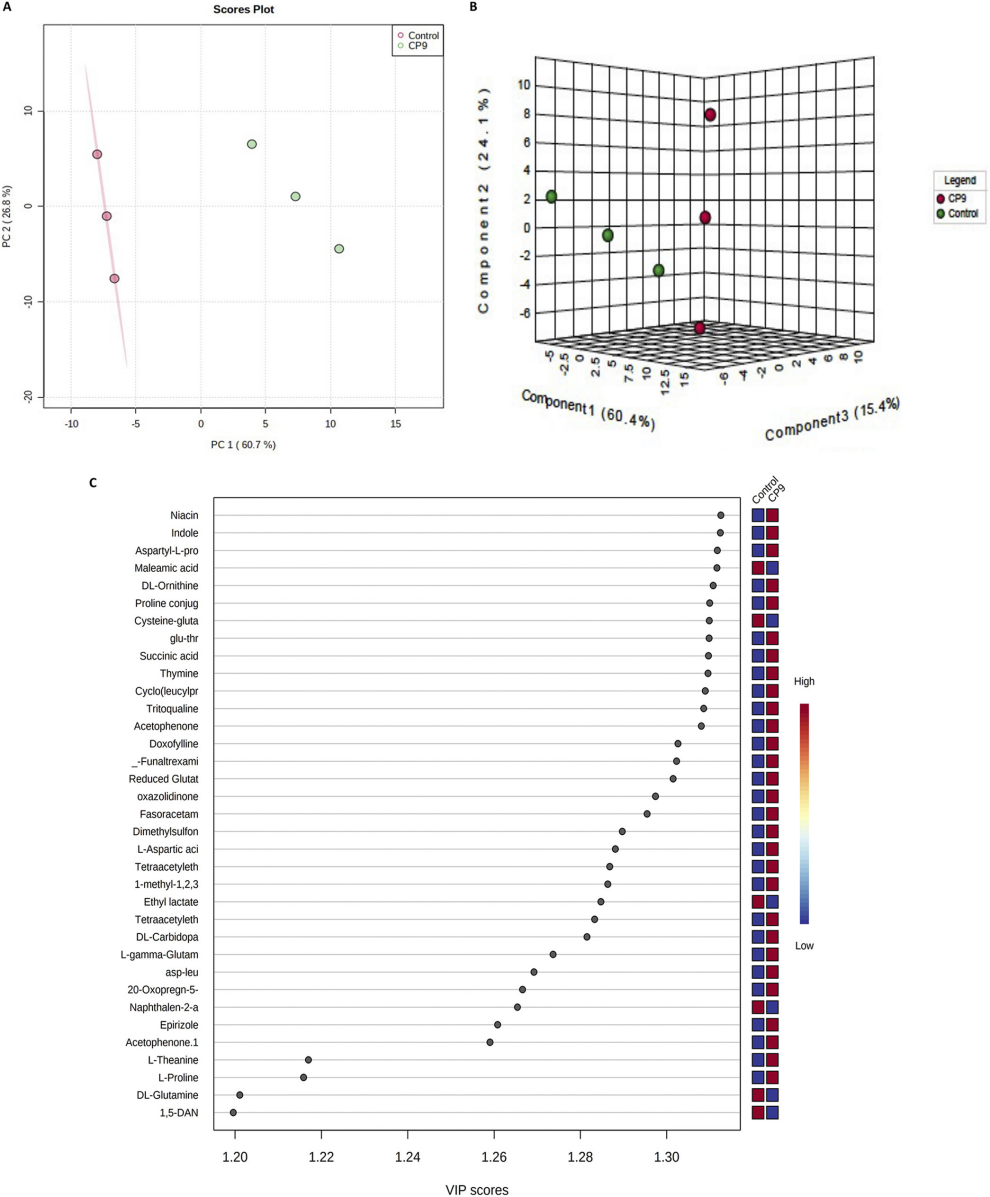
Metabolomic repertoire of IPEC-J2 cells incubated with CP9. Extracellular untargeted metabolomic analyses on the IPEC-J2 cells incubated with CP9, using LC-MS, were performed. (A) PCA scores. Plots are shown with explained variances between the selected PCs. (B) PLS-DA three-dimensional box plot shown with explained variances between the selected PCs. (C) VIP score plot of the important features identified by the PLS-DA model. The colored boxes on the right indicate the relative concentrations of the corresponding metabolite in each group. Statistical analysis was performed using Metaboanalyst (version 5.0) online analysis software with ANOVA testing, with Fisher *post hoc* analysis plus false discovery rate analysis. Features with *P* < 0.05 plus a fold change of >2 were considered significant.

**FIG 11:**
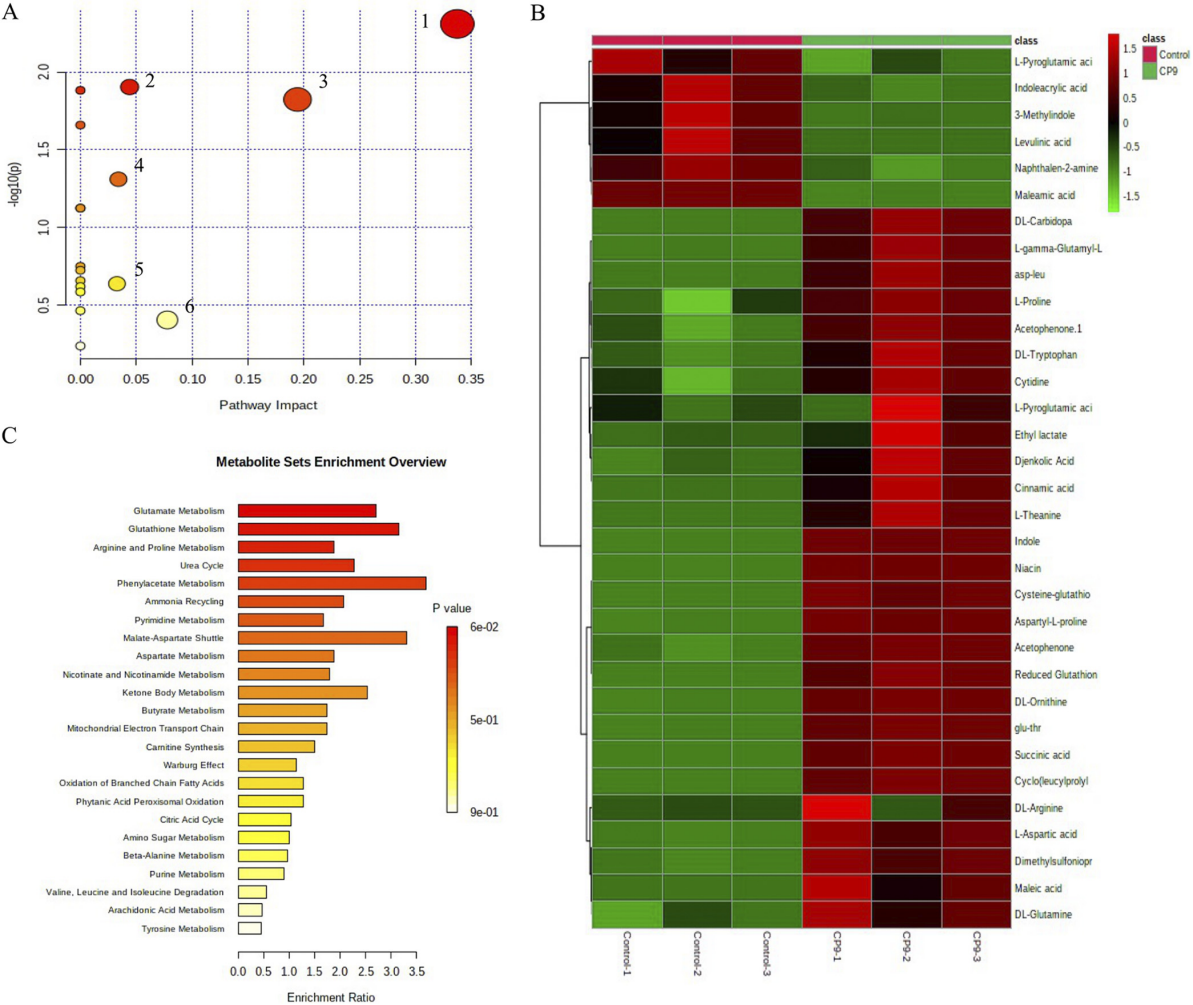
CP9-induced metabolic modulation in IPEC-J2 cells. Biochemical pathway analysis was performed on the significant identified metabolite data using Metaboanalyst (version 5.0) online analysis software. (A) Significantly modulated metabolic pathways determined via pathway impact values calculated from pathway topology analysis: 1, alanine, aspartate, and glutamate metabolism; 2, pyrimidine metabolism; 3, nicotinate and nicotinamide metabolism; 4, glutathione metabolism; 5, the citrate cycle (TCA cycle); and 6, arginine and proline metabolism. (B) Pathway enrichment analysis. (C) Heatmap of the significant differentially expressed metabolites.

To analyze whether CP9 could cause metabolic changes in the IPEC-J2 cells, we performed extracellular untargeted metabolomic analyses on the IPEC-J2 cells incubated with CP9, using liquid chromatography coupled to a mass spectrometer (LC-MS). A total of 113 metabolites were successfully identified (see Table S2 in the supplemental material) which were then statistically analyzed through Metaboanalyst (version 5.0) online analysis software. Incubation with CP9 substantially altered the metabolome of IPEC-J2 cells (see Fig. S1 in the supplemental material). To detect the modulation in the metabolome resulting from the CP9 incubation, we performed principal-component analysis (PCA) on the metabolic profile of CP9-incubated IPEC-J2 cells and no-treatment IPEC-J2 cells. A distinct separation was observed between the metabolomic profiles of no-treatment cells and CP9-incubated cells (Fig. 10A). Component 1 explained 60.7% of the variance, and component 2 explained 26.8% of the variance between the two groups. Analysis of statistically significant (*P *<* *0.05) metabolites identified by Welch’s two-sample *t* test revealed that more metabolites had increased in abundance than had decreased in abundance in the CP9-incubated cells (see Table S3 in the supplemental material). Of the 49 significant metabolites, 37 increased in abundance, and 12 decreased in abundance in response to CP9 administration (see Table S3). To further analyze which metabolites caused maximum variance between the two groups, we performed supervised partial least-squares discriminant analysis (PLS-DA; Fig. 10B). The predictive performance of the model by cross-validation coefficient (Q^2^) was greater than 0.8 (see Fig. S2), suggesting that the models used were of reasonable and acceptable quality. Consistently, a clear separation was observed between the metabolomic profiles of no-treatment cells and CP9-incubated cells via PLS-DA, indicating considerable variation in the IPEC-J2 cells’ metabolite profiles upon incubation with CP9. Furthermore, the variable importance in projection (VIP) score plot in PLS-DA model showed 34 metabolites (VIP score >1.2) responsible for causing maximum separation between the two groups (Fig. 10C). Statistically different metabolites between the groups were then subjected to the pathway impact analysis nodule (in Metaboanalyst version 5) to unravel the metabolic pathways that were being modulated by CP9 in IPEC-J2 cells. The results revealed that CP9 administration caused major changes in 17 metabolic pathways metabolism; out of which, the greatest impact was seen in six major pathways: (i) alanine, aspartate, and glutamate metabolism; (ii) pyrimidine metabolism; (iii) nicotinate and nicotinamide metabolism; (iv) glutathione metabolism; (v) the citrate cycle (the tricarboxylic acid [TCA] cycle); and (vi) arginine and proline metabolism (Fig. 11A). Hierarchical clustering further revealed that metabolites involved in the alanine, aspartate and glutamate metabolism (l-aspartate, dl-glutamine, and succinate) were seen in higher abundance in the CP9-incubated IPEC-J2 cells (Fig. 11B), which may reflect a higher energy demand in the cells (43, 44). Metabolites involved in pyrimidine metabolism (dl-glutamine, cytidine, and thymine) were seen in higher abundance in the CP9-incubated IPEC-J2 cells, which may reflect the higher demand of nucleic acid production owing to cell growth and glucose transport (45, 46). Metabolites involved in the nicotinate and nicotinamide metabolism (dl-tryptophan, l-aspartate, and niacin) were seen in greater abundance in the CP9-incubated IPEC-J2 cells, which may reflect increased energy derivation in the cells for enhanced physiological functions (47, 48). Metabolites involved in glutathione metabolism (cysteine-glutathione disulfide, reduced glutathione, and l-gamma-glutamyl-l-leucine) were seen in greater abundance in the CP9-incubated IPEC-J2 cells, which may reflect a higher antioxidant status and nutrient metabolism in the cells (49), possibly induced by CP9. Finally, metabolites involved in the TCA cycle (succinic acid) and in arginine and proline metabolism (l-proline and dl-arginine) were seen in greater abundance in CP9-incubated IPEC-J2 cells, which may further reflect an increased production of amino acids, cell growth, and energy derivation (50–52) in the CP9-incubated cells.

After hierarchical cluster analysis, we performed metabolite set enrichment analysis by using Metaboanalyst (version 5) to identify detailed pathways that were modulated by the significantly observed metabolites. The results showed that 24 pathways were impacted with CP9 administration (Fig. 11C). Consistently, alanine, aspartate, and glutamate metabolism, pyrimidine metabolism, nicotinate and nicotinamide metabolism, glutathione metabolism, and arginine and proline metabolism were among the top 10 pathways substantially modulated between the two groups. Collectively, these findings indicate that upon the administration of probiotic CP9, the host IPEC-J2 cells undergo notable metabolic changes that may reflect the cytoprotective ability of CP9.

## DISCUSSION

To combat antibiotic resistance in the food chain, new strategies need to be developed. Probiotics may prove to be an effective tool if characterized and developed accurately. We recently isolated and characterized a novel strain of B. subtilis with broad-range antimicrobial activity against enteric pathogens *in vitro*. Here, we used an ETEC infection model in IPEC-J2 cells to analyze the protective role of CP9 as a probiotic candidate and to decipher its mechanism of action. Using a metabolomics approach, we also attempted to decode the metabolic modulation that CP9 may induce in IPEC-J2 cells as part of its cytoprotective role.

ETEC has been shown by various studies *in vitro* and *in vivo* to cause cytotoxicity and cell death, which is primarily driven by its virulence factors, adhesins (for attachment and colonization) and enterotoxins (for toxin secretions) (53–55). Consistently, we found that ETEC substantially affected the cell growth and caused cell cytotoxicity within 4 h of infection. Interestingly, the administration of CP9 pre- or coincubated with ETEC-incubated IPEC-J2 cells attenuated the ETEC-induced cytotoxicity in IPEC-J2 cells. Similar observation has been noted by various studies with B. subtilis-based probiotics (56, 57). Host-microbial interactions are specialized synergistic associations in response to evolutionary cues (58) where intermicrobial survival is defined by competitive exclusion of the opponent by either direct (nutrient depletion) or indirect (toxin secretion) exclusion mechanisms (59). We found that CP9 reduced ETEC attachment to the IPEC-J2 cells, which may explain their competitive exclusion behavior against ETEC. In a mixed microbial environment, Medlock et al. observed distinct differences in the metabolomic profiles of the competitive strain and the negatively affected strain (60). We previously showed in a mixed microbial culture model that CP9 was able to cause metabolic dysregulation in ETEC by competitive acquisition of nutrients, thereby reducing ETEC cell growth (38). Our finding on the lower ETEC cell number observed in a coincubation assay (Fig. 2B) here further supports this notion. Our results suggest that CP9 may exhibit similar competitive behavior in an intestinal model. However, further studies, perhaps via metabolic modeling, must be performed to confirm the production and consumption of the nutrients by each strain in the coculture IPEC cell model.

Nitric oxide plays a significant role in various physiological processes as a secondary messenger and inflammatory modulator (61, 62). While low levels of NO have been shown to be required for maintaining homeostasis in gut, its high levels have been implicated in intestinal inflammation, leading to impaired gut barrier function (63). Nitric oxide is produced by NO synthase by catalyzing the conversion of arginine and oxygen (O_2_) into NO and citrulline (63). Our finding on ETEC-induced nitric oxide production in IPEC-J2 cells was consistent with previous studies where ETEC endotoxins have been shown to significantly increase NO synthase levels (64–66). Consistent with these studies, significant NO levels in our study were detected at 4 h. Interestingly, CP9 significantly lowered NO production in ETEC-infected IPEC-J2 cells, although maintaining a higher-than-normal expression. Our result is consistent with a previous study, wherein probiotic B. subtilis reduced the upregulation of NO synthase and consequent NO levels (17). In addition, previous findings on bactericidal effect of NO may partly explain CP9’s anti-ETEC effect in IPEC-J2 cells (67, 68).

Modulation of the gut immune system has been suggested to be one of the significant modes of action of probiotics (69). With their broad spectrum of pathogen recognition, TLRs play a critical role in innate immunity and consequent signaling pathways, leading to the induction of various proinflammatory cytokines such as TNF-α, IL-6, and IL-8 (70, 71). However, commensals and probiotics experience immunotolerance via a milder activation of associated inflammatory responses (72, 73). For example, a B. subtilis-based probiotic strain was shown to induce a milder immune response via IL-8 activation, which was attenuated when various stressed conditions were tested in B. subtilis-incubated intestinal cells (17). This is consistent in our finding, where despite inducing higher expression of TLR2 and TLR9 mRNA, CP9-incubated cells did not encounter significant immune response (Fig. 4 and 5). On the contrary, ETEC-induced higher mRNA expression of TLRs encountered a significantly higher proinflammatory response (Fig. 4 and 5) in IPEC-J2 cells. Interestingly, CP9-led reduction of proinflammatory cytokines TNF-α, IL-6, and IL-8 in ETEC-infected cells may further suggest its role in negative regulation of TLR signaling pathways (74); however, further analysis must be performed to confirm the specific downstream pathways affected by CP9, such as modulation of the transcription factors nuclear factor kappa light-chain enhancer of activated B cells (NF-κB) and interferon regulatory factors (IRFs). Furthermore, in contrast to previous studies on *Bacillus*-based probiotics, we did not observe higher expression of anti-inflammatory IL-10 (75, 76). However, transcript levels of GM-CSF known for its anti-inflammatory activity (77) was seen significantly higher in the CP9-incubated ETEC-infected cells (Fig. 5). Previous studies have shown that overexpression of GM-CSF alleviates colitis in mice by limiting lipopolysaccharide (LPS)-mediated activation of TLR signaling pathway (78). However, impact of probiotics on the activation of GM-CSF remains unexplored to the best of our knowledge. Our study therefore suggests a novel route of action for *Bacillus*-based probiotic, CP9, where we propose involvement of CP9 in activating GM-CSF and consequently moderating TLR signaling pathways, contributing to alleviating ETEC-induced colitis in IPEC-J2 cells. However, further studies should be performed to confirm the specific TLR pathways being impacted by CP9.

In addition to modulating inflammatory responses, we also observed modulation of host defense peptides gene expression by CP9 (Fig. 6). Although, *Lactobacillus*-based probiotics-induced modulation of HDPs has been described, the impact of *Bacillus*-based probiotics on HDPs remains vastly unexplored (16, 79, 80), including on pigs. Here, we found that CP9 induced significantly higher mRNA expression of MUC1 and PG-1 but not BD3, and this HDP-modulatory property of CP9 was reflected in the homeostatic mRNA expression of these HDPs in CP9-incubated ETEC-infected cells versus ETEC-alone-infected cells. Mucins secreted intestinal epithelium have been previously shown to be degraded by ETEC (56), which was consistent in our study in terms of gene expression. In addition, IL-10 has been shown to stimulate mucin production and attenuate colitis in mice by preventing misfolding of mucin protein (81). However, our findings on CP9 augmentation of mucin transcript expression in ETEC-infected cells did not coincide with increased IL-10 mRNA expression, suggesting the involvement of a different pathway. Our results are also slightly consistent with a previous study wherein a B. subtilis-based probiotic showed increased mRNA expression of mucin 2 (MUC2) in intestinal mucosal cells of broilers (82). Nonetheless, our results suggest that, in addition to competitively excluding ETEC, CP9 may also benefit intestinal cells in avoiding ETEC infection and colonization by inducing or maintaining the secretion of HDPs on mucosal surfaces. However, since our study focused on the modulation in mRNA expression levels, further studies are required to confirm and quantify the protein level expression of these peptides.

Expanding further, our study looked at analyzing impact of CP9 on intestinal barrier function. Intestinal epithelial cells maintain a homeostatic environment by coordinating and regulating permeability, innate and adaptive immunity, and microbial colonization (83). Tight-junction proteins consisting of transmembrane proteins (including claudins, occludin, and intracellular proteins and ZO proteins) tightly control intestinal barrier function and are under constant threat from proinflammatory stimulants (84, 85). Enterotoxigenic E. coli impairs intestinal barrier function *in vitro* and *in vivo* by disrupting the tight-junction proteins claudin-1, occludin, and ZO-1 (86–88). Consistently, our results showed a significant reduction in mRNA expression of tight-junction proteins (Fig. 7) in ETEC-infected cells. However, this deleterious effect was attenuated with the addition of CP9. Our findings on anti-ETEC activity in IPEC-J2 cells may partly explain this beneficial effect. Another possibility is the CP9-mediated activation of GM-CSF, since GM-CSF has been shown to alleviate colitis in mice by elevating the gene expression of ZO-1 (78). Since ETEC infection in intestinal cells also causes structural impairment of the tight-junction proteins (88), further studies are needed to confirm whether CP9 can rescue intestinal cells from this histopathological state.

Maintenance of intestinal epithelial homeostasis is strictly regulated by cellular proliferation in the crypt and apoptotic cell shedding from villus tip. The cell migration from the base of the crypt to the apical epithelial surface sustains matured cell turnover by maintaining rigorous equilibrium between cell death and cell proliferation (42, 89). Increased intestinal cell apoptosis and shedding have been observed in pathological states leading to inflammation led colitis (42, 90–92). ETEC-induced apoptosis has been observed in the IPEC-J2 cells and weaned pigs via activation of caspase-3 (93, 94). In addition, high levels of NO and TNF-α have been directly implicated in the induction of apoptosis and the reduction of epithelial cell turnover (64, 95). Consistent with these studies, along with the higher expression of NO and TNF-α, we observed higher caspase-3 mRNA levels in ETEC-infected IPEC-J2 cells, which were alleviated by addition of CP9. In contrast, GM-CSF stimulates epithelial cell proliferation *in vitro* and *in vivo* (96, 97), and the loss of GM-CSF signaling has been shown to cause ileal barrier dysfunction and increase ileal injury in mice (98). Relatively, our findings on CP9-induced higher GM-CSF and lower caspase-3 transcript levels in ETEC-infected cells suggest a direct role of GM-CSF in probiotic (B. subtilis-based)-induced epithelial cell proliferation. Although *Lactobacillus*-based probiotics have been shown to stimulate epithelial cell proliferation in ETEC-infected intestinal cells and *in vivo* (24, 26, 99), studies on *Bacillus*-based probiotics are rather limited. Hence, results from this study may provide novel insight into the protective mechanisms of *Bacillus*-based probiotics in ETEC-related enteric infections.

Finally, with the help of metabolomics, our study analyzed the impact of CP9 on IPEC-J2 cells. Since the biological impact of the probiotics on the host is mediated through a variety of metabolites produced by these strains, characterization and development of probiotic-led interventions for animal agriculture should include examination of their effects on the metabolome of the host. Metabolomic studies evaluating the effects of probiotics on the intestines of the host are limited. To best of our knowledge, this is the first study to investigate the functional and metabolic interactions of the *Bacillus*-based probiotic and host intestinal cells. The specific aim of this study was to determine the altered metabolites in the CP9-stimulated IPEC-J2 cells to provide a better understanding of the metabolic pathways involved in this interaction. We found that CP9 administration caused substantial changes in 17 metabolic pathway metabolisms; of these, the greatest impact was seen in six major pathways: (i) alanine, aspartate, and glutamate metabolism; (ii) pyrimidine metabolism; (iii) nicotinate and nicotinamide metabolism; (iv) glutathione metabolism; (v) the TCA cycle; and (vi) arginine and proline metabolism (Fig. 10A). Modulation of alanine, aspartate, and glutamate metabolism and of nicotinate and nicotinamide metabolism suggested a direct impact on energy metabolism upon the stimulation of IPEC-J2 cells with CP9. Glutamine has a multifaceted role in cell energy metabolism via the TCA cycle, the biosynthesis of nucleotides, glutathione (GSH), and glycolysis (100). Previous studies have shown that glutamine stimulates intestinal cell proliferation by activation of mitogen-activated protein kinases and augmenting epidermal growth factor (101, 102). Restriction of glutamine levels has been shown to impair cellular replication, tight-junction proteins, and permeability in intestinal cells (102–104), which are reversed by the addition of glutamine. In addition, growth factor stimulation can increase the rate of glycolysis for supporting cellular proliferation (105). Since, alanine, aspartate, and succinate also increase the energy status in the cells via the TCA cycle and glycolysis, our findings on the greater abundance of aspartate, glutamine, and succinate in CP9-stimulated cells suggest that CP9 may enhance the energy status of IPEC-J2 cells, which could aid increased cell proliferation. This notion is further supported by higher abundance of nicotinamide (NAD) metabolites (dl-tryptophan and niacin), which are directly involved in the regulation of energy homeostasis for cell growth and survival (106, 107). Nicotinamide supplementation has been shown to rejuvenate intestinal stem cells from aged mice by modulating the NAD/SIRT1/mTORC1 axis (108). Probiotic- and microbiota-enhanced NAD biosynthesis has been reported in microbiota-depleted mice (109, 110). A protective effect of niacin has been demonstrated in ulcerative colitis via prostaglandin D_2_-mediated D prostanoid receptor 1 activation in dextran sulfate sodium-challenged mice (111). Taken together, our findings suggest the involvement of CP9 in modulating energy metabolism in IPEC-J2 cells. This is particularly beneficial with respect to intestinal cells, since the energy requirement is very high due to physiological function and cell renewal (112).

Relatedly, enhanced cell proliferative status requires augmented nucleotide synthesis (113). Glutamine acts a nitrogen donor for the *de novo* synthesis of nucleotides (114). Our findings from the pathway enrichment and impact analysis suggested the involvement of l-glutamine in modulating pyrimidine metabolism in CP9-stimulated IPEC-J2 cells. This was further accompanied by a greater abundance of pyrimidine metabolites, cytidine, and thymine in CP9-stimulated IPEC-J2 cells. Since we observed an increase in cell proliferation in ETEC-infected IPEC-J2 cells upon CP9 stimulation, a greater abundance of the nucleotides in CP9-stimulated IPEC-J2 cells further supports the notion of CP9-induced cell proliferation in IPEC-J2 cells. However, further studies are needed to confirm these effects and to determine the specific proliferative pathway impacted by CP9 stimulation.

Glutamine has been shown to suppress NF-κB pathway-induced inflammation in a rodent model of colitis (115) and in LPS-treated enterocytes of neonatal piglets (116). In addition, glutamine displays antiapoptotic activity via modulation of caspase activation (116, 117), the production of antioxidant GSH (118), and enhancement of heat shock proteins in intestinal epithelial cells (119, 120). This is particularly interesting since we saw a decrease in apoptosis via lower caspase-3 activity in CP9-treated ETEC cells. Our observations on higher cell proliferation and lower caspase-3 activity, together with our findings on the higher abundance of metabolites from glutathione metabolism, therefore suggest that CP9 may enhance the antioxidant status of host IPEC-J2 cells, which may be mediated by glutamine and glutamine-regulated inflammation.

In conclusion, our study describes a protective role of a novel probiotic B. subtilis isolated from sub-Saharan camels in swine epithelial cells *in vitro.* Our study suggests that in IPEC-J2 cells the novel B. subtilis CP9 confers protection against ETEC. This cytoprotection may be attributed to CP9’s ability to moderate the ETEC-induced inflammation, strengthen the intestinal epithelial barrier function, reduce apoptosis, and improve cell proliferation, possibly by metabolic modulation. However, metabolomic results in our study should be interpreted cautiously, since only three data points for each treatment were used for this study. In addition, since our study only focused on these interactions in an ETEC infection model, further studies need to be performed using other infection models to assess and confirm whether CP9’s protective activity is pathogen specific. Findings from our study provide important cues for future studies to further characterize and determine the use of CP9 either as a prophylactic or as a treatment for postweaning diarrhea and for the development of next-generation probiotics for use in animal agriculture and humans.

## MATERIALS AND METHODS

### Bacterial culture.

Bacillus subtilis CP9 was isolated previously from sub-Saharan camel feces and characterized in our lab at the University of Guelph, Guelph, Ontario, Canada (37). Fresh stocks of the strain were stored and preserved in a −80°C Ultra-low-temperature Freezer (Thomas Scientific). CP9 was grown in Luria-Bertani (LB) liquid broth medium at 37°C, in shaking incubator under aerophilic conditions.

The E. coli strain used in our study was F4-expressing enterotoxigenic E. coli (ETEC), serotype K88. The strain was acquired from the Animal Health Lab, University of Guelph, and was grown in LB medium at 37°C in a shaking incubator under aerophilic conditions. As an additional control, a commercially available B. subtilis strain (CS) was used for comparative analysis. The strain was grown in LB liquid broth medium at 37°C in a shaking incubator under aerophilic conditions.

### Cell line and culture conditions.

The swine intestinal epithelial cell line IPEC-J2, originally derived from jejuna of neonatal piglets was acquired from the American Type Culture Collection (Cedarlane, Burlington, Ontario, Canada). IPEC-J2 cells were cultured in a 1:1 mixture of Dulbecco modified Eagle medium/Ham’s nutrient medium F-12 (DMEM/F-12) supplemented with 10% fetal bovine serum (FBS), 1% penicillin-streptomycin solution (Gibco/Fisher Scientific, Mississauga, Ontario, Canada) under 5% CO_2_ in a 95% air atmosphere with 90% humidity at 37°C. The culture medium was replaced every 3 days, and the cells were split after reaching 80% confluence with 0.25% trypsin-EDTA (Gibco/Fisher Scientific).

For bacterial coincubation experiments, supplemented DMEM/F-12 nutrient medium was replaced after 24 h with CO_2_-independent medium (Gibco/Fisher Scientific) supplemented with l-glutamine (GlutaMAX supplement; Gibco/Fisher Scientific) and 10% FBS.

### ETEC infection model in IPEC-J2 cells. (i) Pre- and coincubation assays.

ETEC infection model was created as previously described (121) with minor modifications. Briefly, IPEC-J2 cells were seeded at a density of 4 × 10^5^ cells per well in six-well tissue culture plates (Corning; Fisher Scientific). After 24 h of incubation, the medium was replaced with incomplete CO_2_-independent medium supplemented with l-glutamine and FBS. For the preincubation assay, IPEC-J2 cells were pretreated with phosphate-buffered saline (PBS)-washed 10^8^ CFU of CP9 or CS/well for 3 h and then stimulated by the addition of 10^8^ CFU of ETEC/well. The cells were incubated in a 37°C incubator for 4 h. Similarly, for the coincubation assay, IPEC-J2 cells were incubated with 10^8^ CFU of CP9 or CS/well, along with 10^8^ CFU of ETEC/well, followed by incubation in a 37°C incubator for 4 h. The morphology of the cells was assessed by using Cytation 5 Cell Imaging Multi-Mode Reader (BioTek Instruments, Winooski, VT) at ×20 magnification, using a 100-μm scale in bright-field mode. After removal of the medium, cells were washed twice with PBS and collected using 0.25% trypsin-EDTA. The numbers of viable cells were counted by using a standard trypan blue assay with a TC20 Automated Cell Counter (Bio-Rad Laboratories, Ltd., Mississauga, Ontario, Canada).

### (ii) Cell adhesion inhibition assay.

The cell adhesion assay was performed as previously described (122) with minor modifications. Briefly, IPEC-J2 cells were seeded at a density of 2 × 10^5^ cells per well in six-well tissue culture plates (Corning) and grown under 5% CO_2_ in a 95% air atmosphere with 90% humidity at 37°C. After 24 h of incubation, the medium was replaced with incomplete CO_2_-independent medium supplemented with l-glutamine and FBS, followed by incubation in a 37°C incubator for 2 h. After an initial adjustment for 2 h, PBS-washed (twice) 10^8^ CFU of CP9, ETEC, and CP9-ETEC/mL together were added to IPEC-J2 cells in respective wells. The cells were cultured for an additional 3 h. The culture medium was removed, and the cells were washed twice with PBS to remove unbound bacterial cells. IPEC-J2 cells were collected using 0.25% trypsin-EDTA incubation for 2 min. Adherent bacteria were serial diluted by 10-fold in LB medium and plated on LB agar plates. The numbers of CP9 and ETEC colonies were counted after aerobic incubation for 24 h. CP9 and ETEC were enumerated in LB and MacConkey agar plates, respectively.

### Nitric oxide production measurement in IPEC-J2 cell line.

The induction of NO by IPEC-J2 cells upon treatment with ETEC and CP9 was assessed as previously described (123), with minor modifications. Briefly, IPEC-J2 cells were seeded in a 96-well tissue culture plate until 80% confluence. The cells were then stimulated with 10^8^ CFU/mL ETEC or CP9 monocultures or 10^8^ CFU/mL ETEC-CP9 cocultures, followed by incubation at 37°C for 3 h. Nitric oxide production induced by different treatments was measured by using a Griess reagent system (Promega, Madison, WI), which measures nitrite as a primary breakdown product of NO. Manufacturer’s instructions were followed to perform this assay. Briefly, 100 μL of the cell culture mixture was transferred to a new 96-well tissue culture plate, and 100 μL of Griess reagent was added to the each well; samples were incubated for 15 min and protected from light. NO was then detected by measuring the absorbance at 540 nm by using a spectrophotometer with a Cytation 5 Cell Imaging Multi-Mode Reader (BioTek Instruments). The results were expressed as nitrite concentrations (μM) produced, by plotting a standard curve.

### Cell proliferation assay.

The impact of CP9 on the proliferation of IPEC-J2 cells during ETEC infection was assessed as previously described (124), with minor modifications. Briefly, IPEC-J2 cells were seeded in a 96-well plate at a density of 3 × 10^4^ cells/well, followed by incubation at 37°C for 24 h. Spent medium was then replaced with CO_2_-independent medium without antibiotics. IPEC-J2 cells were stimulated with 10^8^ CFU of ETEC/mL for 2 h at 37°C. The spent medium was then removed, and cells were incubated with fresh medium containing 10^8^ CFU of CP9/mL at 37°C for an additional 6 h. The final volume in each well was 100 μL. Cells with or without FBS were used as controls. Cell proliferation was measured by using a CCK-8 kit (Bimake, Houston, TX) according to the manufacturer’s instructions. Briefly, 10 μL of the CCK-8 reagent was added to each well, followed by incubation at 37°C for 2 h. Metabolically active cells in proliferation were counted by measuring the absorbance at 450 nm using a Cytation 5 Cell Imaging Multi-Mode Reader (BioTek Instruments).

### Caspase-3 activity assay in IPEC-J2 cells.

The activity of caspase-3 was determined as previously described (125) by using a caspase-3 activity kit (Sigma-Aldrich, Oakville, Ontario, Canada), which is based on the ability of caspase-3 to hydrolyze the acetyl-Asp-Glu-Val-Asp *p*-nitroanilide (Ac-DEVD-pNA) substrate into a yellow formazan *p*-nitroaniline (pNA) moiety. Briefly, a coincubation assay was performed as described above. After 4 h of incubation, cell lysates were prepared by centrifuging cells at 12,000 × *g* for 15 min at 4°C. Cell lysates (10 μL) were added to each well in a flat-bottom, 96-well culture plate, followed by incubation with 10 μL of Ac-DEVD-pNA overnight at 37°C. Controls used in the assay included the following: no treatment, a caspase-3 positive control (provided with kit), and a caspase-3 inhibitor (provided with the kit). The caspase-3 activity was determined by measuring the absorbance value of released pNA at 405 nm by using a Cytation 5 Cell Imaging Multi-Mode Reader.

### Real-time PCR.

A coincubation assay with ETEC infection was performed as described above. Briefly, IPEC-J2 cells were seeded at a density of 4 × 10^5^ cells per well in six-well tissue culture plates. After 24 h of incubation, the medium was replaced with incomplete CO_2_-independent medium supplemented with l-glutamine and FBS. IPEC-J2 cells were then infected with 10^8^ CFU of ETEC/well, along with 10^8^ CFU of CP9/well, and incubated in a 37°C incubator for 4 h. The cells were collected, and the total RNA was isolated from the cells using a Norgen total RNA purification kit (Norgen BioTek, Thorold, Ontario, Canada). The RNA yield and quality were assessed spectrophotometrically with a NanoDrop 8000 spectrophotometer (Thermo Fisher Scientific). An extracted RNA sample was reverse transcribed to complementary DNA using an iScript Reverse Transcription Supermix kit (Bio-Rad Laboratories, Ltd., Berkeley, CA). Quantitative real-time-PCR (qPCR) was conducted using a Bio-Rad CFX Connect real-time system. Primers were designed using the Primer-BLAST tool (National Center for Biotechnology Information) and synthesized by Integrated DNA Technologies, Guelph, Ontario, Canada. Primer information is listed in Table S1 in the supplemental material. The efficiencies of the primers were calculated by using CFX Manager Software (Bio-Rad Laboratories, Ltd.). Real-time quantitative PCR was performed at 95°C, activating the enzyme for 2 min; at 95°C, with denaturing for 5 s; and then at 60°C, with annealing for 20 s. Reactions were repeated for 40 cycles. The efficiency of each primer set was calculated by performing qPCR, and only primer sets with an efficiency between 90 and 110% were selected for the downstream gene expression analysis. The product size of each primer set was verified by agarose gel electrophoresis. Samples were assessed in triplicate. The relative mRNA expression was analyzed by normalizing the cycle threshold (*C_T_*) values of the target genes to the *C_T_* values of the housekeeping gene encoding GAPDH. The results are presented as the fold change calculated using the 2^−ΔΔ^*^CT^* method.

### Metabolomic analyses: Sample preparation and LC-MS procedure.

To determine the metabolic impact of CP9 on IPEC-J2 cells, a coculture experiment was performed wherein CP9 was incubated with IPEC-J2 cells for 5 h. After the end of the incubation period, spent supernatants from negative-control cells and CP9-incubated cells were collected and filter sterilized using Fisher 0.22-μm-pore-size nylon filters. Nutrient medium (CO_2_-independent medium) was used as a blank. The samples were immediately frozen in liquid nitrogen and stored in a –80°C Ultra-Freezer. Samples were packed in dry ice and shipped to the BioZone Mass Spectrometry Facility in the Chemical Engineering Department, University of Toronto, for metabolite extraction and LC-MS analysis (courtesy of metabolomics specialist Robert Flick). Analysis was performed as previously described (38). Briefly, metabolites were vacuum dried after protein precipitation and resuspended in appropriate starting solvent for each chromatography analysis. A Thermo Scientific Ultimate 3000 UHPLC (Thermo Fisher Scientific, Waltham, MA) equipped with Hypersil Gold C_18_ column (50 mm × 2.1 mm, 1.9 μm) or a Phenomenex Luna NH_2_ column (150 mm × 2mm, 3 μm) were then used to analyze the samples. Temperature at 40°C and a flow rate of 300 μL min^−1^ were set for the column. Water and acetonitrile containing 0.1% formic acid were used as eluents. Two separate gradients were performed for each column. A gradient at 5% B (1 min), a linear gradient at 98% B (6 min), maintained at 98% B (3 min), returned to 5% B (0.5 min), and finally a reequilibration at 5% B (4.5 min) (total runtime, 15 min) were performed for the C_18_ column. A gradient at 90% B (1 min), a linear gradient at 5% B (4 min), maintained at 5% B (8 min), returned to 90% B (1 min), and finally a reequilibration at 90% B (6 min) (total runtime, 20 min) were performed for the Luna NH_2_ column. Liquid samples (10 μL) were loaded onto the autosampler. The autosampler temperature was kept at 10°C. A Q-Exactive Orbitrap mass spectrometer (Thermo Fisher Scientific) equipped with a heated electrospray ionization (HESI II) probe was used for compound detection. The system was operated in negative and positive ionization modes to generate spectra. After generating the raw peaks, we processed the untargeted metabolomic data (raw signal exacting, data baseline filtering, peak identification, and integration) and metabolite detection (KEGG and BioCyc database) using a differential analysis software package (Compound Discoverer 2.1; Thermo Scientific).

### Statistical analyses.

All experiments were performed in three biological replicates, and data are presented as means ± the standard errors of the mean (SEM). For gene expression analysis, experiments were performed in triplicate (*n *=* *3), and data are presented as means ± the SEM. Data were analyzed using Prism v7.0 (GraphPad Software, Inc., San Diego, CA) using one-way or two-way analysis of variance (ANOVA) with the Tukey *post hoc* test. A *P* value of <0.05 was considered significant for all statistical tests.

Metaboanalyst (version 5.0) online analysis software (https://www.metaboanalyst.ca; accessed 20 May 2021) was used to analyze metabolomic data. Briefly, samples were first normalized to the internal control and blank control. Processed data were then submitted to Metabolanalyst software for performing univariate and multivariate analyses. PCA and PLS-DA, combined with *t* tests (two-sample *t* tests and Wilcoxon rank sum tests) analyses, were used to screen the significantly differential metabolites. A *P *value of <0.05 was considered significant for all statistical tests. The model was evaluated by the cross-validation (leave-one-out cross-validation) method, using Q^2^ as a performance measure. Clustering and pathway analysis was performed by generating a heat map using Euclidean distances and complete linkages with *t* test results.

### Data availability.

The data presented in this study are available on request from the corresponding author.
