# Supplementary material for: A Novel Probiotic Bacillus subtilis Strain Confers Cytoprotection to Host Pig Intestinal Epithelial Cells during Enterotoxic Escherichia coli Infection
Source: Microbiol Spectr. 2022 Jun 23;10(4):e01257-21. doi: 10.1128/spectrum.01257-21 (PMC9430607; doi:10.1128/spectrum.01257-21)
Supplement: Supplemental file 1 — Supplemental material. Download spectrum.01257-21-s0001.pdf, PDF file, 0.6 MB [file spectrum.01257-21-s0001.pdf]

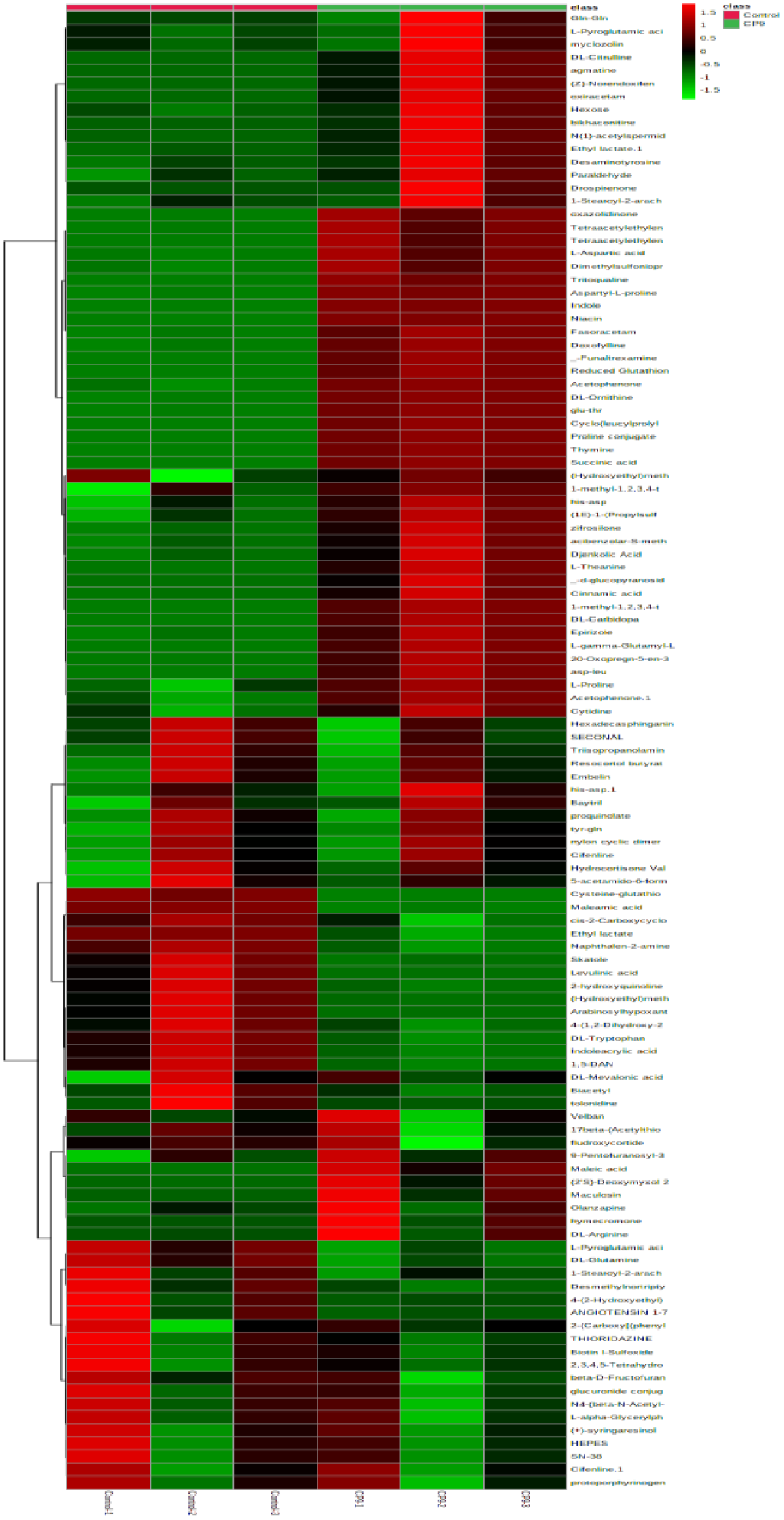

class
Control
CP9
1.5
1
0.5
0
-0.5
-1
-1.5
Gln-Gln
L-Pyroglutamic aci
myclozolin
DL-Citrulline
agmatine
(Z)-Norendoxifen
oxiracetam
Hexose
bikhaconitine
N(1)-acetylspermid
Ethyl lactate.1
Desaminotyrosine
Paraldehyde
Drospirenone
1-Stearoyl-2-arach
oxazolidinone
Tetraacetylethylen
Tetraacetylethylen
L-Aspartic acid
Dimethylsulfoniopr
Triloquadine
Aspartyl-L-proline
Indole
Niacin
Fasoracetam
Doxofylline
_-Fursultiamine
Reduced Glutathion
Acetophenone
DL-Ornithine
glu-thr
Cyclo(leucylprolyl
Proline conjugate
Thymine
Succinic acid
(Hydroxyethyl)meth
1-methyl-1,2,3,4-t
his-asp
(1E)-1-(Propylsulf
zifrosilone
acibenzolar-S-meth
Djenkolic Acid
L-Theanine
_-d-glucopyranosid
Cinnamic acid
1-methyl-1,2,3,4-t
DL-Carbidopa
Epirizole
L-gamma-Glutamyl-L
20-Oxopregn-5-en-3
asp-leu
L-Proline
Acetophenone.1
Cytidine
Hexadecasphinganin
SECONAL
Triisopropanolamin
Resorcinol butyrat
Embelin
his-asp.1
Bayinl
proquinolate
tyr-gln
nylon cyclic dimer
Cifenline
Hydrocortisone Val
5-acetamido-6-form
Cysteine-glutathio
Maleamic acid
cis-2-Carboxycyclo
Ethyl lactate
Naphthalen-2-amine
Skatole
Levulinic acid
2-hydroxyquinoline
(Hydroxyethyl)meth
Arabinosylhypoxant
4-(1,2-Dihydroxy-2
DL-Tryptophan
Indoleacrylic acid
1,5-DAN
DL-Mevalonic acid
Biacetyl
tolonidine
Velban
17beta-(Acetylthio
fludroxycortide
9-Pentofuranosyl-3
Maleic acid
(2'S)-Deoxymyxol 2
Maculosin
Olanzapine
hymecromone
DL-Arginine
L-Pyroglutamic aci
DL-Glutamine
1-Stearoyl-2-arach
Desmethylnortripty
4-(2-Hydroxyethyl)
ANGIOTENSIN 1-7
2-(Carboxy[(phenyl
THIORIDAZINE
Biotin l-Sulfoxide
2,3,4,5-Tetrahydro
beta-D-Fructofuran
glucuronide conjug
N4-(beta-N-Acetyl-
L-alpha-Glyceryiph
(+)-syringaresinol
HEPES
SN-38
Cifenline.1
protoporphyrinogen
Control-1
Control-2
Control-3
CP9-1
CP9-2
CP9-3

Supplement Figure 1. Global metabolomic profile of IPEC-J2 cells incubated with CP9 as identified by LC-MS platform. Differential metabolite pattern between negative control and CP9 incubated IPEC-J2 samples are seen via heat map generated using Metaboanalyst (version 5.0) online analysis software (www.metaboanalyst.ca, Accessed 20 May 2021).

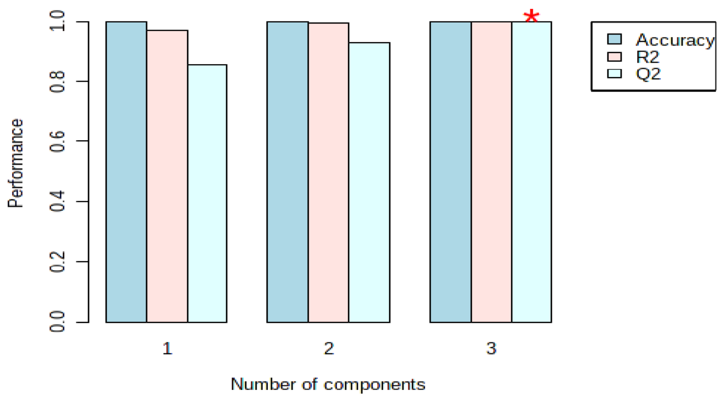

Supplement Figure 2. Cross Validation classifier for predicting PLS-DA model accuracy using different number of components. Red star indicates the best classifier. Supervised multivariate analysis on the identified metabolites performed using Metaboanalyst (version 5.0) online analysis software (www.metaboanalyst.ca, Accessed 20 May 2021).

Table S1. Genes and primer sequences used for quantitative real time PCR.

Gene	Primer sequence
TLR2	F-ACGTATCCATCAATGAACACTGC<br>R-GTCCGTTAAGGGTGCAGTCA
TLR4	F-GCCATCGCTGCTAACATCATC<br>R-CTCATACTCAAAGATACACCATCGG
TLR9	F-CACGACAGCCGAATAGCAC<br>R-GGGAACAGGGAGCAGAGC
TNFα	F-GGCCCAAGGACTCAGATCAT<br>R-GGCATACCCACTCTGCCATT
IL-6	F-CCCTGAGGCAAAAGGGAAAGAA<br>R-CTCAGGTGCCCCAGCTACAT
IL-8	F-TTCGATGCCAGTGCATAAATA<br>R-CTGTACAACCTTCTGCACCCA
GM-CSF	F-GGCTGTGATGAATGAAACCGTA<br>R-GTACAGGTTCAGGCGAGTCT
IL-10	F-TGCATCCACTTCCCAACCAG<br>R-GGCAACCCAGGTAACCCTTA
BD3	F-CCTTCTCTTTGCCTTGCTCTT<br>R-GCCACTCACAGAACAGCTACC
MUC1	F-CTCTGCTCAGCCTGGGTCT<br>R-GCTACATAGGATGGTAGGCA
PG-1	F-TCTCTTGGTCACAGGTTCA<br>R-GACACAGACGCAGAACCTA
Claudin-1	F-AGCTATGGCCAACGCGG<br>R-TGCTTGCAAAGTGGTGTTCAG
Occludin	F-ATCAACAAAGGCAACTCT<br>R-GCAGCAGCCATGTACTCT

Zona occludens-1	F-GAGTTTGATAGTGGCGTT<br>R-GTGGGAGGATGCTGTTGT
GAPDH	F-AGCAATGCCTCCTGTACCAC<br>R-AAGCAGGGATGATGTTCTGG

Table S2. Metabolomic profiles of no treatment IPEC-J2 cells and CP9-incubated IPEC-J2 cells as successfully identified by LC-MS platform and their contribution to the first principal component (PC1) and the second principal component (PC2).

METABOLITE	PC1	PC2
DL-Tryptophan	-0.10774	-0.073517
Indoleacrylic acid	-0.10568	-0.081215
nylon cyclic dimer	0.028691	-0.17645
Acetophenone	0.11717	0.041168
Desaminotyrosine	0.10689	-0.065307
(Hydroxyethyl)methacrylate	0.058765	0.10517
Thymine	0.11832	0.029734
1-Stearoyl-2-arachidonoyl-sn-glycero-3-phosphate	-0.071439	0.03077
Succinic acid	0.11829	0.029928
2-hydroxyquinoline	-0.1009	-0.091112
Indole	0.11615	0.038175
Cifenline	0.026811	-0.17726
Tetraacetylethylenediamine	0.10815	0.058416
Niacin	0.11641	0.038018
(2'S)-Deoxymyxol 2'-alpha-L-fucoside	0.077781	0.097531
4-(2-Hydroxyethyl)phenyl hydrogen sulfate	-0.079847	0.073849
bikhaconitine	0.10827	-0.04674
(Hydroxyethyl)methacrylate.1	-0.097116	-0.10006
4-(1,2-Dihydroxy-2-propanyl)-1-methyl-1,2-cyclohexanediol	-0.099042	-0.081565
_-Funaltrexamine	0.11945	0.023053
1,5-DAN	-0.1062	-0.080734
Ethyl lactate	-0.12006	-0.018542
1-methyl-1,2,3,4-tetrahydro-beta-carboline-3-carboxylic acid	0.096775	-0.076062
Hydrocortisone Valerate	0.014423	-0.17385
Hexadecasphinganine	-0.036522	-0.17154
Proline conjugate	0.11821	0.03038
Naphthalen-2-amine	-0.11509	-0.051954
Fasoracetam	0.12005	0.016007
Resocortol butyrate	-0.0068145	-0.18133
5-acetamido-6-formamido-3-methyluracil	-0.0042125	-0.16868
Maculosin	0.068049	0.10356

**Cifenline.1**	-0.032736	0.17503
**20-Oxopregn-5-en-3-yl hydrogen sulfate**	0.12054	0.0062744
**Skatole**	-0.10057	-0.0974
**Cyclo(leucylprolyl)**	0.11847	0.028972
**_-d-glucopyranoside conjugate**	0.11719	-0.021463
**glucuronide conjugate**	-0.074138	0.13945
**cis-2-Carboxycyclohexyl-acetic acid**	-0.11402	-0.023629
**Levulinic acid**	-0.099488	-0.097881
**Cinnamic acid**	0.1186	-0.016484
**Tritoqualine**	0.11373	0.046419
**Maleic acid**	0.092214	0.083008
**acibenzolar-S-methyl**	0.11792	-0.031861
**Biacetyl**	-0.080214	-0.10098
**2-{Carboxy[(phenylacetyl)amino]methyl}-5,5-dimethyl-1,3-thiazolidine-4-carboxylic acid**	-0.014366	0.15905
**hymecromone**	0.054405	0.11
**his-asp**	0.11315	-0.056982
**N(1)-acetylspermidine**	0.11094	-0.039875
**Embelin**	-0.0012836	-0.18157
**SECONAL**	-0.041832	-0.16914
**(Z)-Norendoxifen**	0.11336	-0.034478
**Drospirenone**	0.099834	-0.06177
**DL-Arginine**	0.050829	0.10756
**Acetophenone.1**	0.11641	0.042408
**DL-Citrulline**	0.113	-0.035322
**oxiracetam**	0.11372	-0.033499
**Paraldehyde**	0.10974	-0.069617
**L-Aspartic acid**	0.10827	0.062018
**L-Pyroglutamic acid**	0.087727	-0.044388
**DL-Ornithine**	0.11798	0.030086
**HEPES**	-0.05598	0.15529
**L-Proline**	0.11233	0.043957
**Cysteine-glutathione disulfide**	-0.11647	-0.029587
**L-Pyroglutamic acid.1**	-0.10146	-0.0093365
**Ethyl lactate.1**	0.11038	-0.048576
**Tetraacetylethylenediamine.1**	0.10738	0.061205
**Olanzapine**	0.040115	0.084417

**Desmethylnortriptyline**	-0.095702	0.073688
**Hexose**	0.10768	-0.029511
**Epirizole**	0.12066	6.1115e-05
**THIORIDAZINE**	-0.07071	0.12539
**oxazolidinone**	0.11049	0.053793
**Biotin l-Sulfoxide**	-0.064894	0.13913
**L-Theanine**	0.11962	-0.0079264
**DL-Glutamine**	-0.10247	-0.010193
**L-gamma-Glutamyl-L-leucine**	0.12053	0.0087407
**DL-Mevalonic acid**	-0.0078917	-0.091803
**myclozolin**	0.086303	-0.052779
**(1E)-1-(Propylsulfanyl)-1-propene**	0.11681	-0.041952
**Maleamic acid**	-0.1158	-0.043829
**Dimethylsulfoniopropionate**	0.1086	0.063388
**asp-leu**	0.12049	0.0082543
**Gln-Gln**	0.083884	-0.076327
**proquinolate**	0.011276	-0.18078
**1-Stearoyl-2-arachidonoyl-sn-glycero-3-phosphate.1**	0.095814	-0.093308
**his-asp.1**	0.058865	-0.14757
**tyr-gln**	0.022697	-0.17795
**Doxofylline**	0.11949	0.020342
**Reduced Glutathione**	0.11954	0.022127
**SN-38**	-0.056407	0.15422
**(+)-syringaresinol**	-0.048683	0.16402
**tolonidine**	-0.070456	-0.12418
**Velban**	-0.021093	0.15014
**agmatine**	0.11276	-0.035817
**Arabinosylhypoxanthine**	-0.097103	-0.10179
**Triisopropanolamine**	-0.021378	-0.17885
**Aspartyl-L-proline**	0.11545	0.040005
**9-Pentofuranosyl-3,9-dihydro-1H-purine-2,6-dione**	0.058193	0.012781
**2,3,4,5-Tetrahydroxypentanal**	-0.059339	0.13599
**17beta-(Acetylthio)estra-1,3,5(10)-trien-3-ol acetate**	-0.038963	0.046855
**N4-(beta-N-Acetyl-D-glucosaminyl)-L-asparagine**	-0.073806	0.14353
**1-methyl-1,2,3,4-tetrahydro-beta-carboline-3-carboxylic acid.1**	0.12032	0.014239
**protoporphyrinogen**	-0.051778	0.16386

**beta-D-Fructofuranosyl 4-O-dodecanoyl-alpha-D-glucopyranoside**	-0.091111	0.11901
**zifrosilone**	0.11907	-0.0218
**DL-Carbidopa**	0.12045	0.011799
**ANGIOTENSIN 1-7**	-0.084588	0.066669
**Djenkolic Acid**	0.11744	-0.031738
**L-alpha-Glycerylphosphorylcholine**	-0.069463	0.14868
**Baytril**	0.066652	-0.15099
**fludroxycortide**	-0.064135	0.077224
**Cytidine**	0.11263	0.034304
**glu-thr**	0.11828	0.029067

Table S3. Significant change in the metabolites emerging in CP9 incubated IPEC-J2 cells relative to no treatment IPEC-J2 cells as assessed by Welch's two-sample *t*-test.

METABOLITE	t.stat	p.value	-log10(p)	FDR
**Niacin**	-750.8	1.8882e-11	10.724	2.1337e-09
**Indole**	-147.83	1.2561e-08	7.901	7.0967e-07
**Aspartyl-L-proline**	-57.055	5.6505e-07	6.2479	1.9982e-05
**Maleamic acid**	53.937	7.0733e-07	6.1504	1.9982e-05
**DL-Ornithine**	-38.423	2.7405e-06	5.5622	6.1935e-05
**Proline conjugate**	-31.86	5.7853e-06	5.2377	8.6868e-05
**Cysteine-glutathione disulfide**	31.271	6.2318e-06	5.2054	8.6868e-05
**glu-thr**	-31.066	6.3979e-06	5.194	8.6868e-05
**Succinic acid**	-30.199	7.1619e-06	5.145	8.6868e-05
**Thymine**	-29.667	7.6875e-06	5.1142	8.6868e-05
**Cyclo(leucylprolyl)**	-26.911	1.1335e-05	4.9456	0.00011644
**Tritoqualine**	-25.628	1.3769e-05	4.8611	0.00012965
**Acetophenone**	-24.037	1.7768e-05	4.7504	0.00015444
**Doxofylline**	-16.163	8.5713e-05	4.067	0.00068832
**_-Funaltrexamine**	-15.904	9.137e-05	4.0392	0.00068832
**Reduced Glutathione**	-15.314	0.00010607	3.9744	0.0007491
**oxazolidinone**	-13.053	0.00019882	3.7015	0.0013216
**Fasoracetam**	-12.273	0.00025314	3.5966	0.0015892
**Dimethylsulfoniopropionate**	-10.581	0.00045138	3.3455	0.0026845
**L-Aspartic acid**	-10.217	0.00051721	3.2863	0.0029223
**Tetraacetylethylenediamine**	-9.9484	0.00057336	3.2416	0.0030507
**1-methyl-1,2,3,4-tetrahydro-beta-carboline-3-carboxylic acid.1**	-9.8582	0.00059394	3.2263	0.0030507
**Ethyl lactate**	9.5641	0.00066768	3.1754	0.0032803
**Tetraacetylethylenediamine.1**	-9.3164	0.0007388	3.1315	0.0034785
**DL-Carbidopa**	-9.0352	0.00083126	3.0803	0.0037573
**L-gamma-Glutamyl-L-leucine**	-8.0352	0.001302	2.8854	0.0056585
**asp-leu**	-7.5917	0.001615	2.7918	0.006759
**20-Oxopregn-5-en-3-yl hydrogen sulfate**	-7.3595	0.001816	2.7409	0.0073289
**Naphthalen-2-amine**	7.2612	0.0019103	2.7189	0.0074437
**Epirizole**	-6.9117	0.0022988	2.6385	0.0086589
**Acetophenone.1**	-6.7894	0.0024575	2.6095	0.008958

**L-Theanine**	-4.951	0.0077559	2.1104	0.027181
**L-Proline**	-4.9185	0.0079377	2.1003	0.027181
**DL-Glutamine**	4.5388	0.010507	1.9785	0.034338
**1,5-DAN**	4.504	0.01079	1.967	0.034338
**DL-Tryptophan**	4.486	0.01094	1.961	0.034338
**zifrosilone**	-4.3729	0.011941	1.923	0.035618
**L-Pyroglutamic acid.1**	4.3411	0.012243	1.9121	0.035618
**Indoleacrylic acid**	4.3358	0.012293	1.9103	0.035618
**Maleic acid**	-4.2517	0.01314	1.8814	0.03712
**Cinnamic acid**	-4.2178	0.013501	1.8696	0.037159
**(1E)-1-(Propylsulfanyl)-1-propene**	-4.1896	0.013811	1.8598	0.037159
**Skatole**	3.9707	0.016529	1.7817	0.043437
**Cytidine**	-3.9176	0.017283	1.7624	0.043619
**cis-2-Carboxycyclohexyl-acetic acid**	3.9116	0.01737	1.7602	0.043619
**acibenzolar-S-methyl**	-3.8449	0.018382	1.7356	0.045155
**_-d-glucopyranoside conjugate**	-3.6932	0.020961	1.6786	0.048843
**Levulinic acid**	3.6838	0.021134	1.675	0.048843
**Djenkolic Acid**	-3.6813	0.021179	1.6741	0.048843
